# Novel Thiourea and Oxime Ether Isosteviol-Based Anticoagulants: MD Simulation and ADMET Prediction

**DOI:** 10.3390/ph17020163

**Published:** 2024-01-28

**Authors:** Marcin Gackowski, Mateusz Jędrzejewski, Sri Satya Medicharla, Rajesh Kondabala, Burhanuddin Madriwala, Katarzyna Mądra-Gackowska, Renata Studzińska

**Affiliations:** 1 Department of Toxicology and Bromatology, Faculty of Pharmacy, L. Rydygier Collegium Medicum in Bydgoszcz, Nicolaus Copernicus University in Torun, A. Jurasza 2 Street, 85089 Bydgoszcz, Poland; 2 Department of Organic and Physical Chemistry, Faculty of Pharmacy, Medical University of Warsaw, Banacha 1 Street, 02093 Warsaw, Poland; mateusz.jedrzejewski@wum.edu.pl; 3 Doctoral School, Medical University of Warsaw, Żwirki i Wigury 81 Street, 02093 Warsaw, Poland; 4 Department of Pharmaceutical Chemistry, Faculty of Pharmacy, M. S. Ramaiah University of Applied Sciences, Bengaluru 560054, Karnataka, India; srisatyamedicharla@gmail.com (S.S.M.); burhanmadri53@gmail.com (B.M.); 5 Thapar Institute of Engineering and Technology, Patiala 147004, Punjab, India; kondabalarajesh94@gmail.com; 6 Department of Geriatrics, Faculty of Health Sciences, L. Rydygier Collegium Medicum in Bydgoszcz, Nicolaus Copernicus University in Torun, Skłodowskiej Curie 9 Street, 85094 Bydgoszcz, Poland; katarzyna.madra@cm.umk.pl; 7 Department of Organic Chemistry, Faculty of Pharmacy, L. Rydygier Collegium Medicum in Bydgoszcz, Nicolaus Copernicus University in Torun, A. Jurasza 2 Street, 85089 Bydgoszcz, Poland; rstud@cm.umk.pl

**Keywords:** anticoagulants, activated blood coagulation factor X (FXa) inhibitors, isosteviol, molecular dynamics (MD) simulation, thrombosis, ADMET prediction

## Abstract

Activated blood coagulation factor X (FXa) plays a critical initiation step of the blood-coagulation pathway and is considered a desirable target for anticoagulant drug development. It is reversibly inhibited by nonvitamin K antagonist oral anticoagulants (NOACs) such as apixaban, betrixaban, edoxaban, and rivaroxaban. Thrombosis is extremely common and is one of the leading causes of death in developed countries. In previous studies, novel thiourea and oxime ether isosteviol derivatives as FXa inhibitors were designed through a combination of QSAR studies and molecular docking. In the present contribution, molecular dynamics (MD) simulations were performed for 100 ns to assess binding structures previously predicted by docking and furnish additional information. Moreover, three thiourea- and six oxime ether-designed isosteviol analogs were then examined for their drug-like and ADMET properties. MD simulations demonstrated that four out of the nine investigated isosteviol derivatives, i.e., one thiourea and three oxime ether ISV analogs, form stable complexes with FXa. These derivatives interact with FXa in a manner similar to Food and Drug Administration (FDA)-approved drugs like edoxaban and betrixaban, indicating their potential to inhibit factor Xa activity. One of these derivatives, E24, displays favorable pharmacokinetic properties, positioning it as the most promising drug candidate. This, along with the other three derivatives, can undergo further chemical synthesis and bioassessment.

## 1. Introduction

Direct FXa inhibitors that are currently commercially available include rivaroxaban, apixaban, edoxaban, and betrixaban, which are an important group of anticoagulants most widely prescribed for the prophylaxis and treatment of thrombotic disorders as a safer alternative to warfarin [1,2]. Their popularity in the market is due to their potency for anticoagulation and their specificity for FXa when administered orally [3]. Xabans, introduced after 2011, work by binding selectively and reversibly to FXa. They are approved for stroke prevention in patients with nonvalvular atrial fibrillation (AF), the prophylactic treatment of deep vein thrombosis (DVT) leading to pulmonary embolism (PE), and the treatment of DVT and PE to mitigate the risk of recurrence. AF guidelines consider non-vitamin-K antagonist oral anticoagulants to be the preferred choice of anticoagulants to prevent stroke in patients with AF [4]. At present, NOACs are commonly used in a number of countries. In this connection, Eliquis^®^ (apixaban) was ranked sixth amongst the 10 best-selling drugs in the world, which generated almost USD 12 billion in 2022 alone [5]. On the one hand, NOACs have a more favorable efficacy–safety ratio and a predictable anticoagulant effect, eliminating the need for routine coagulation monitoring in comparison to vitamin-K anticoagulants [4]. On the other hand, it is widely understood that the safe and effective use of new anticoagulants necessitates an understanding of key patient factors, such as their age, liver and renal function, and pregnancy status. It is also important to consider any other medications the patient may be taking, as many of these new anticoagulants are substrates of P-glycoprotein and may interact with other drugs, which can pose a potential risk. By taking these factors into account, healthcare providers can better assess the benefits and risks of these new anticoagulants for their patients [6]. It should be emphasized that patients who take NOACs are at a higher risk of bleeding compared to those who are not on anticoagulants. The risk of bleeding in patients taking NOACs can be categorized into intracranial hemorrhage, gastrointestinal bleeding, genitourinary bleeding, and respiratory bleeding. However, studies have suggested that NOACs are associated with a lower risk of gastrointestinal bleeding in comparison to warfarin [2]. In light of the above, the potential restrictions on the use of anticoagulants encourage the continued search for new, safer, and effective anticoagulants acting as FXa inhibitors.

Rivaroxaban and apixaban were the first FXa inhibitors approved by the FDA in 2011 and 2012, respectively. The next molecule, which received marketing authorization in 2015, was edoxaban and then betrixaban two years later [1]. The pivotal role of FXa in the blood-coagulation pathway makes it an attractive target for anticoagulant drug development [7]. That is why more and more researchers have designed, synthesized, and evaluated the biological activity of numerous potential FXa inhibitors based on different scaffolds. Molecules investigated for FXa inhibition between 2015 and 2022 comprise, among others, anthranilate, pyrazolopyridone, isoxazolopyrimidinone, diaminobenzamide, triazole, dioxolamide [3], N-1,2,3-triazole, and propargyltetrahydroquinoline derivatives [8] and some FXa inhibitors from natural products [3]. For some of the discovered molecules, the inhibition rate of FXa and anticoagulant activity in vitro or in vivo were very close or even better than those for approved drugs. The abovementioned pyrazolopyridone and triazole derivatives demonstrated low FXa inhibitory activity. Anthranilate and triazole derivatives are considered to be structures worthy of further development and, in particular, should undergo in vivo investigation. Hence, it is crucial to continue efforts aimed at creating and developing novel FXa inhibitors.

**Figure 1:**
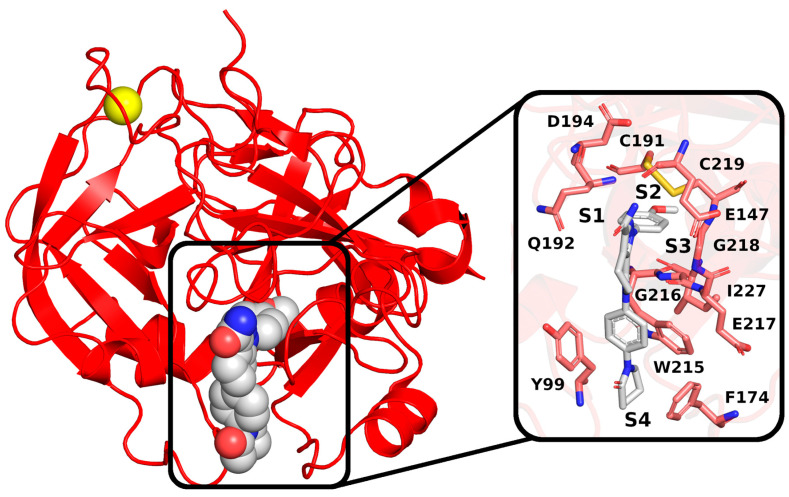
The crystal structure of *H. sapiens* FXa (red) in complex with a calcium cation (yellow) and an inhibitor; PDB code: 2P16. The inset shows the binding pocket, with key residues highlighted. The binding pocket comprises four subpockets: S1 (constructed by C191, Q192, D194, and I227), S2 (C219), S3 (E147 and G218), and S4 (Y99, F174, W215, G216, and E217).

Recently almost forty isosteviol(ISV) analogs bearing two different moieties, i.e., thiourea [9] and oxime ether [10] isosteviol derivatives as FXa inhibitors, were designed based on structural knowledge derived from quantitative structure–activity relationship (QSAR) studies and molecular docking simulation. For both groups of ISV-like compounds, promising predicted FXa inhibitory activity was described, and also, interactions with the active site of FXa were revealed. According to the molecular docking results obtained, the fused polycyclic region of the designed ISV compounds is involved in molecular interaction with the S4 pocket and the derivative part with the S1 and the S2 pocket of FXa (Figure 1). Such interactions are characteristic of novel synthetic FXa inhibitors [11]. Molecular docking as an alternative to experimental methods has made it possible to select from among a large group of the designed FXa inhibitors, with a potential nine drugs that may undergo laboratory tests (Table 1).

Computational prediction of the ligand–protein complex structure is crucial for successful structure-based drug design. Although many computational docking methods exist, the accuracy of predictions is still rather limited [12]. In a docking calculation, the software searches for the binding pose of the ligand molecule in the active site of the target protein, and, subsequently, the binding affinity is evaluated with an empirical scoring function. This process often faces a significant challenge due to the limited accuracy of the empirical score. The score is based on a single predicted structure, which may not take into account essential dynamic effects. To address this issue, it has been suggested that MD simulation could be utilized to enhance the prediction of binding free energy, provide complementary information, and, in turn, increase the efficiency of drug development.

The objective of this study was to use an in silico pharmacoinformatics approach to validate and prioritize potentially active candidate compounds from newly designed thiourea and oxime ether ISV derivatives. MD simulations were conducted for 100 ns as a continuation of previous molecular docking studies [9,10] to evaluate the validity of the FXa–ligand binding pose predictions and to furnish additional information. Subsequently, the compounds were screened for ADME/drug-likeness properties. This approach was implemented to facilitate the identification of promising compounds for further studies, i.e., chemical synthesis and biological evaluation.

## 2. Results and Discussion

### 2.1. Molecular Dynamic (MD) Simulation and Analysis

#### 2.1.1. ISV Derivative Complexes with FXa Exhibit Variable Stability

**Figure 2:**
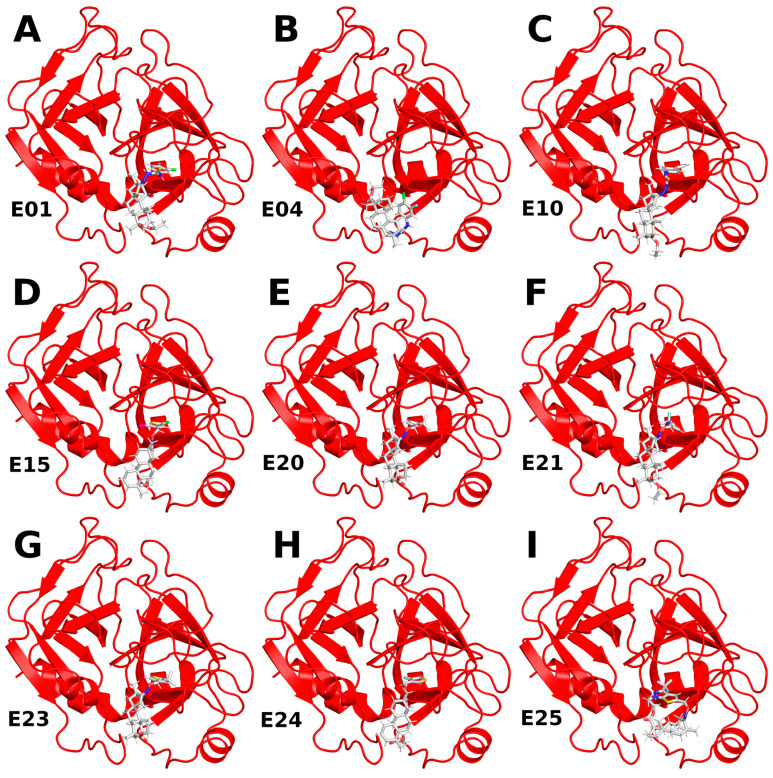
Structures of complexes between ISV derivatives and FXa (red) obtained through docking (**A**–**I**). These structures served as the initial configurations for the subsequent MD simulations.

**Figure 3:**
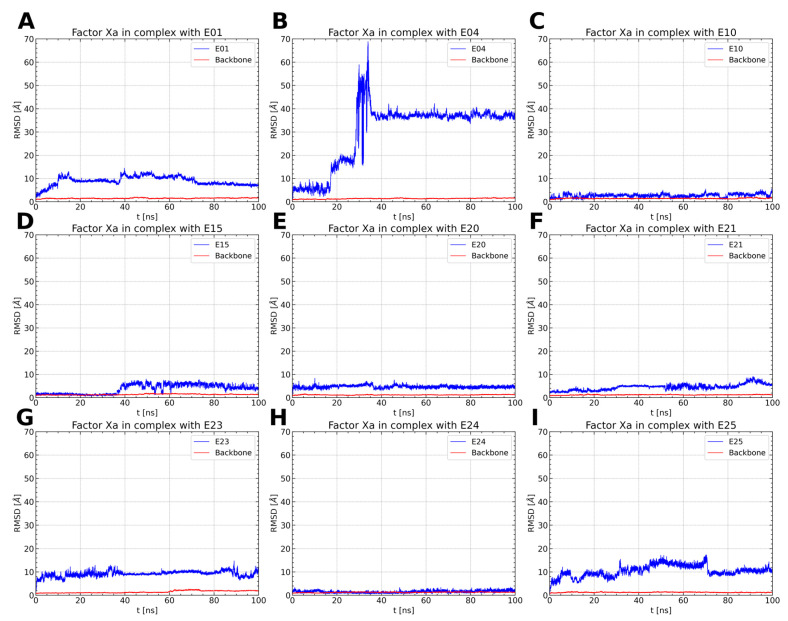
RMSD values of protein backbone (red) and ligand (blue) for the simulation of an FXa complex with different ligands (**A**–**I**). While the RMSD for the protein backbone remains stable with consistently small values across all simulations, the RMSD of the ligand varies between simulations, indicating distinct stability levels of the complexes.

**Figure 4:**
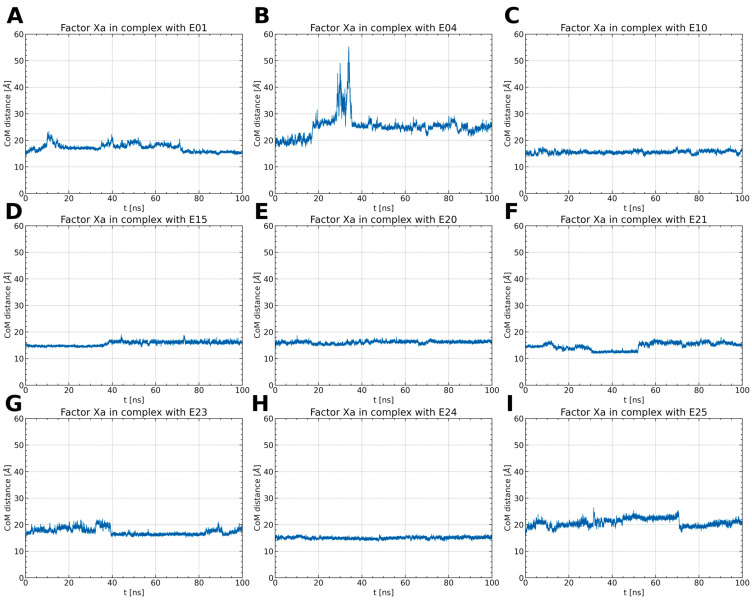
The distance between the CoM of the ligand and the CoM of FXa throughout the simulations (**A**–**I**). Typically, the distance remains around 15 to 20 Å, except for the FXa complex with E04. In this case, after 15 ns, the distance increases rapidly, stabilizing at 25 Å. This observation implies the dissociation of the ligand and its potential rebinding at a different site.

To examine the stability of FXa complexes with designed ISV derivatives, 100 ns MD simulations were carried out. The structures obtained from docking studies, previously published, were utilized as the initial structures for the MD simulations (Figure 2) [9,10]. The stability of the complex was assessed by calculating the RMSD of the protein main chain and the RMSD of the ligand, with the initial structure from the MD simulation serving as the reference structure. In all trajectories, the RMSD of the protein’s main chain remains stable at approximately 2 Å, indicating the stability of the protein structure (Figure 3). Additionally, the distance between the center of mass (CoM) of the ligand and the CoM of FXa was also measured (Figure 4). However, the RMSD fluctuations of the ligand and the distance between the CoM of the ligand and the CoM of the protein are trajectory dependent, suggesting different stabilities of the complexes involving different ISV derivatives.

Among the thiourea derivatives of ISV, only compound E10 forms a stable complex with FXa, as indicated by the stable distance between the centers of mass (Figure 4C) and the stable RMSD of the ligand throughout the simulation (Figure 3C). In contrast, stable complexes with FXa are not formed by the other two compounds, E01 and E04. The RMSD of the ligand increases rapidly in both cases (Figure 3A,B). Moreover, during simulations for E04, the ligand was observed to disassociate and bind to another part of the protein. This observation is supported by the rapid increase in the distance between the centers of mass after approximately 15 ns of simulation time, followed by stabilization around 35 ns (Figure 4B). For the E01 derivative simulations, the distance between the centers of mass fluctuates between 15 and 20 Å (Figure 4A). The fluctuation in center of mass distances, coupled with significant changes in RMSD, implies rapid alterations in the ligand-binding mode without dissociation from the binding site. For the oxime ether derivatives of ISV, stable complex formation was observed in compounds E15, E20, and E24. The RMSD of the ligand fluctuates around low values for the derivatives E20 and E24 (Figure 3E,H). However, in the case of compound E15, a noticeable spike in RMSD values occurs around 40 ns into the simulation (Figure 3D). Similarly, the distance between the centers of mass is stable in simulations for the E20 and E24 derivatives, while it increases after 40 ns in simulations for E01 (Figure 4D,E,H). These changes in RMSD value and CoM distance are associated with a conformational change of the ligand. For other oxime ether derivatives, significant changes in RMSD are accompanied by minor alterations in the distance between the centers of mass. This suggests that, despite substantial changes in the binding pose of ligands, they maintain some interactions with the protein throughout the simulation. In the following, the conformational changes of the ligand and the interactions between the ligand and FXa are analyzed for the stable complexes, namely E10 for the thiourea derivatives of ISV and E15, E20, and E24 for the oxime ether derivatives.

**Figure 5:**
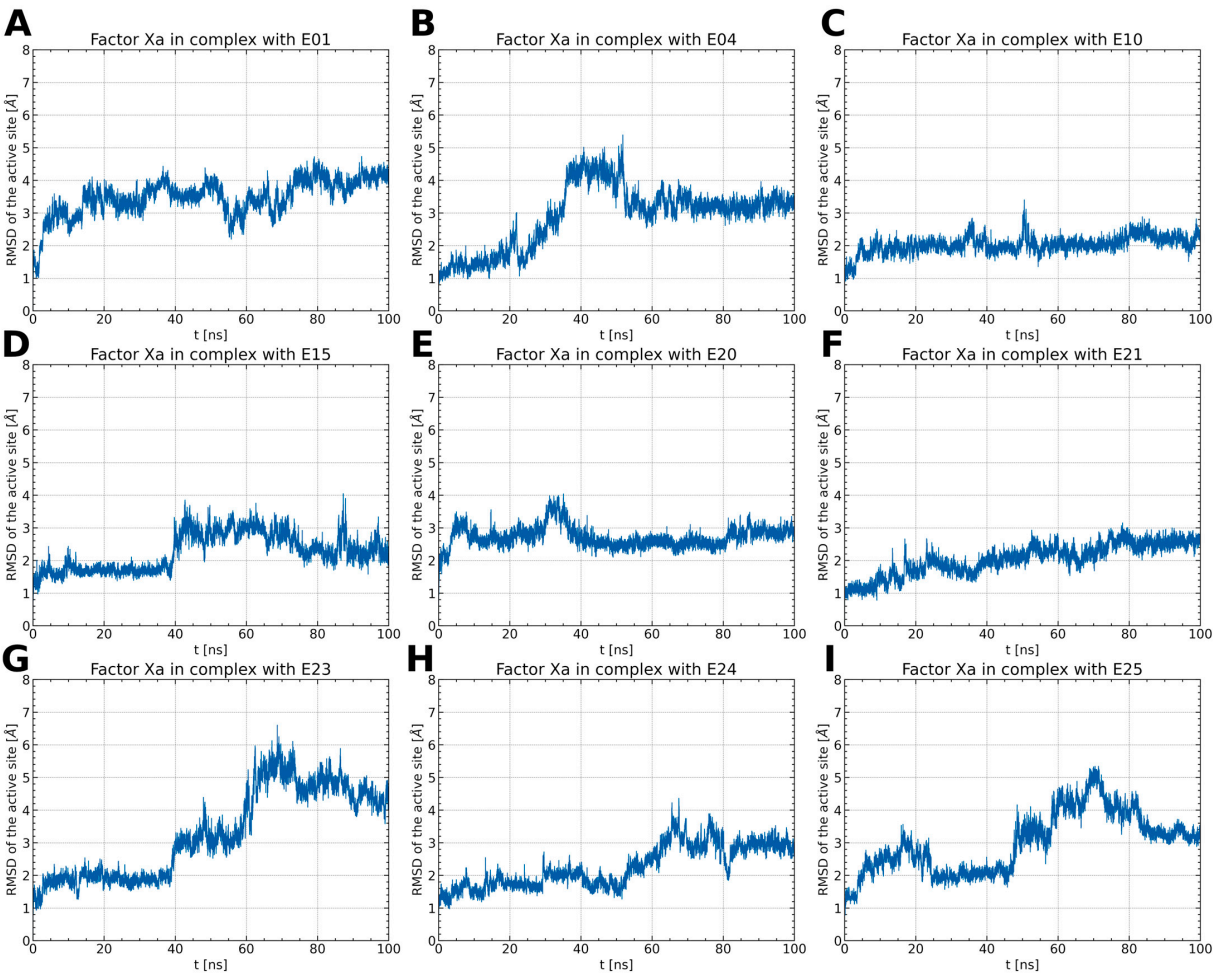
The RMSD changes of amino acids forming the binding pocket (Y99, E147, F174, C191, Q192, D194, W215, G216, E217, G218, C219, and I227) (**A**–**I**). The RMSD for FXa complexes with E10, E20, and E21 remains relatively stable, with low values ranging from 2 to 3 Å. In contrast, other complexes show larger RMSD changes, indicating diverse mobility of the ligand-binding site, dependent on the specific type of ligand bound.

The conformational dynamics of the ligand-binding site were investigated by assessing the RMSD of the amino acids forming the binding pocket (Figure 5). In complexes of ISV derivatives with FXa, characterized by notable changes in the ligand’s RMSD (E01, E04, E23, and E25), significant alterations in the RMSD of the residues building the binding pocket were observed (Figure 5A,B,G,I). This supports the hypothesis that changes in the ligand pose may be attributed to conformational variations in the binding pocket. Interestingly, in the trajectory of the FXa complex with E21, the RMSD of the residues in the binding pocket does not undergo significant changes, in contrast to the previously mentioned complexes (Figure 5F). This suggests that alterations in the pose of E21 may not be due to conformational changes in the binding pocket but rather from suboptimal docking results.

For stable complexes of FXa with ISV derivatives (E10, E15, E20, and E24), the RMSD of the residues constituting the binding pocket remains within the range of 2 to 3 Å and does not exhibit abrupt changes (Figure 5C–E,H). In the case of the E15 complex, there is a noticeable rise in the RMSD of the binding pocket around 40 ns, correlating with a simultaneous increase in the RMSD of the ligand. This suggests that the conformational change in the binding pocket influences the pose of the ligand. Interestingly, in the case of E24, the RMSD of the binding pocket increases around 50 ns of simulation time (Figure 5H). However, this alteration in the configuration of amino acid residues does not impact the conformation of the ligand.

#### 2.1.2. ISV Derivatives Exhibit Different Conformational Dynamics

**Figure 6:**
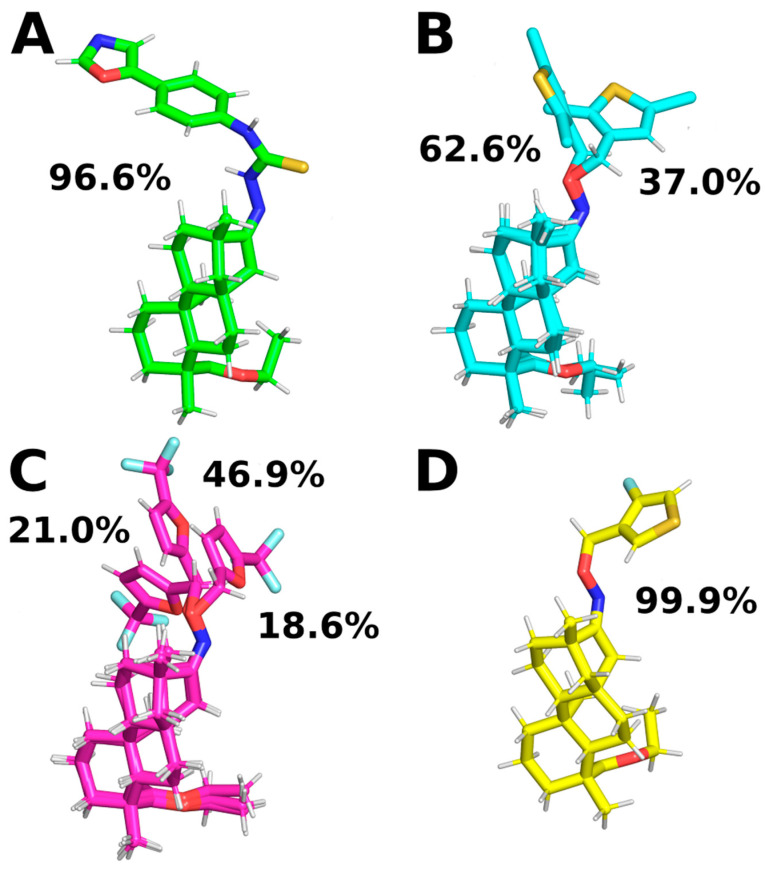
Representative ligand conformations bound to FXa. Conformations were chosen through ligand clustering using the GROMOS algorithm with a cutoff of 1.0 Å, based on frames sampled every 100 ps. Conformations found in less than 5% of frames are not shown for clarity. (**A**): E10, (**B**): E15, (**C**): E20, and (**D**): E24.

The ISV derivatives within the studied complexes with FXa exhibited diverse conformational dynamics. To analyze the conformational changes of the ligands, a cluster analysis was employed to obtain the most representative conformations for each ISV derivative. During MD simulations of the two ISV derivatives, E10 and E24, it was observed that the ligand primarily adopts a single conformation, constituting 96.6% and 99.9% of the simulation time, respectively (Figure 6A,D). The most frequent conformation of the ligand in the FXa complex with E10, derived from simulations, exhibits minor deviation from the ligand pose obtained through docking, serving as the initial geometry for MD simulations (Table 2). For E24, the difference between the ligand conformation obtained through docking and the one most frequently observed in MD simulations is even smaller, with an RMSD of 0.45 Å for the heavy ligand atoms.

In the case of the MD simulations for the other two complexes, namely E15 and E20, the conformations adopted by the ligands are more varied. For the E15 derivative, two different conformations were observed, with varying similarities to the ligand pose obtained by docking. Interestingly, the conformation occurring for 37.0% of the simulation time is the most similar to the ligand pose obtained by docking, while the most common conformation differs from it (Table 2). This suggests that the ISV derivative E15 undergoes conformational changes during molecular dynamics simulations, indicating that the conformation obtained by docking is not stable. Indeed, there is a notable increase in the RMSD value of the E15 ligand (Figure 3D), indicating a change in the ligand’s conformation from the initially docked state to a conformation corresponding to the most frequent cluster. This change in the RMSD of the ligand is associated with a simultaneous change in the RMSD of the binding pocket, suggesting that a change in the conformation of the binding site causes a change in the conformation of the ligand. During MD simulations of the E20 complex with FXa, the ligand was found to adopt three distinct conformations, each characterized by variations in the conformation of the derivative part (Figure 6C). Interestingly, the graph of RMSD changes reveals a lack of abrupt changes in the RMSD of the ligand, suggesting more frequent and smoother transitions between the three conformations (Figure 3E). This observation indicates increased flexibility in the structure of the ligand. Contrary to the simulations for E15, in the case of E20, the most frequent ligand conformation is also the one closest to the conformation obtained through docking (Table 2).

#### 2.1.3. ISV Derivatives Exhibit a Similar Pattern of Interaction to FDA-Approved FXa Inhibitors

**Figure 7:**
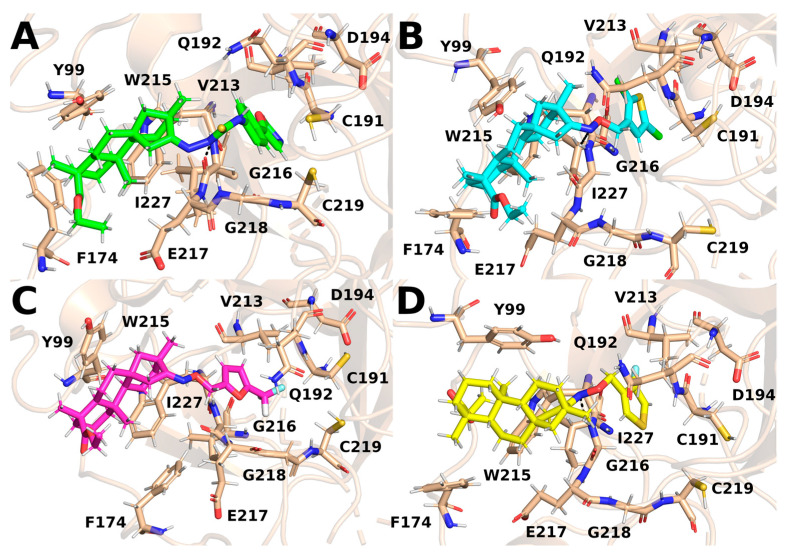
Snapshots from MD simulations for the most representative conformations of the ligands shown in Figure 6. The stick representation depicts the side chains of key residues and ligands. Hydrogen bonds are shown with dashed lines. (**A**): E10, (**B**): E15, (**C**): E20, and (**D**): E24.

To characterize the interactions stabilizing the complex between ISV derivatives and FXa, snapshots for the representative ligand conformations were analyzed. The binding pocket of FXa can be divided into four subpockets: S1, S2, S3, and S4. The designed ISV derivatives interact with factor Xa in a similar manner. The derivative part interacts with subpocket S1, composed of residues C191, Q192, D194, and I227, and subpocket S2, consisting of residues G218, C219, and E147. The polycyclic part of the derivative fits into subpocket S4. The main interactions of ISV derivatives with subpocket S4 involve hydrophobic interactions with aromatic amino acid residues, such as Y99, F174, and W215, as well as hydrogen bonding with G216 (Figure 7).

The key amino acid residue involved in hydrogen-bond formation with ISV derivatives is G216 (Table 3). Among the four analyzed derivatives, only E20 does not form hydrogen bonds with G216 or other amino acids in the binding pocket. Hydrogen bonding between ISV derivatives and G216 persisted for a significant portion of the trajectories for E10, E15, and E24. The average distance between donor and acceptor remained within a few Å in the simulations, with a low standard deviation, indicating high stability in the interactions with this amino acid residue. Ether oxime derivatives of ISV (E15 and E24) functioned as hydrogen-bond acceptors in interactions with G216, whereas E10, a thiourea derivative, acted as a hydrogen-bond donor. In addition, during MD simulations, E15 formed a hydrogen bond with the Y99 residue. Although this bond was present for a significant portion of the simulation time (26.23%), the average distance between the donor and the acceptor was larger than typical hydrogen bonds, and it exhibited a large standard deviation. These characteristics suggest that this hydrogen bond is unstable and breaks during MD simulations. The breaking of this hydrogen bond is associated with a conformational change of the ligand, transitioning from the initially docked state to a conformation corresponding to the most frequent cluster.

The ISV derivatives interact with amino acid residues comprising the S4 subpocket (Table 4). For each of the four ISV derivatives, hydrophobic interactions with the side chains of Y99 and W215 were present for almost the entire length of the simulation. However, the derivatives exhibited variability in their interactions with the F174 residue. In particular, interactions with F174 were present for more than 80% of the simulation time for the FXa complexes with E10 and E24, while for complexes E15 and E20, this interaction was observed for 57.40% and 26.10%, respectively. Interestingly, hydrophobic interactions with the F174 residue persisted for the majority of the simulation time in FXa complexes with ligands that exhibited less conformational variation, namely E10 and E24, while occurring less frequently for the others. This observation suggests that stronger ligand–F174 interactions may stabilize the ligand’s conformation, modifying its flexibility and potentially influencing the overall stability of the complex.

The interactions stabilizing the FXa complex with thiourea and ISV ether oxime derivatives closely resemble those with FDA-approved FXa inhibitors, such as edoxaban and betrixaban, currently in clinical use [13]. Similar to edoxaban and betrixaban, ISV derivatives interact with crucial residues forming the S4 pocket, including Y99, F174, and W215. The significance of residues Y99 and F174 in ligand binding is underscored by the destabilizing impact of Y99A and F174A mutations on the binding of apixaban to the FXa active site [14]. Additionally, the involvement of these residues, along with W215, in the binding of FXa inhibitors has been reported by published experimental studies [15]. Moreover, except for E20, they engage in the formation of hydrogen bonds with G216, a key residue in the binding of FXa inhibitors. Notably, none of the tested compounds forms hydrogen bonds with the G218 amino acid residue, part of the S2 pocket, distinguishing them from FXa complexes with edoxaban and betrixaban. This unique property sets isosteviol derivatives apart from these two drugs. The capability to form stable complexes with FXa, creating interactions similar to FDA-approved inhibitors, positions ISV derivatives as promising drug candidates and a solid foundation for further research, including experimental studies.

### 2.2. ADMET prediction

**Figure 8:**
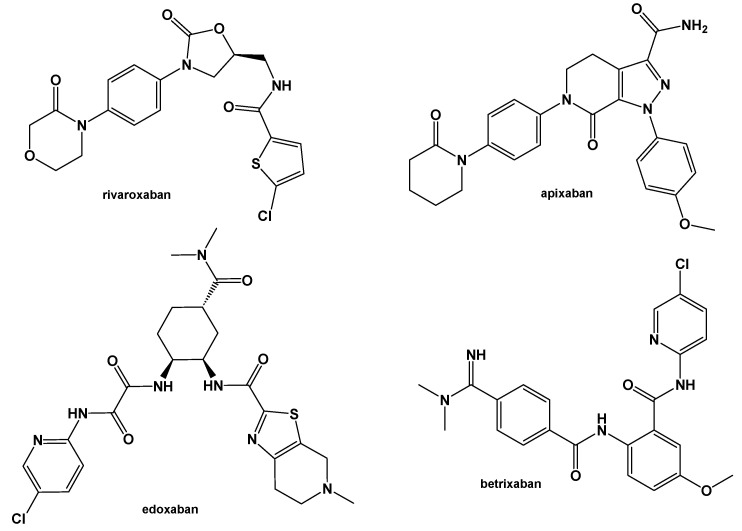
Chemical structures of FDA-approved FXa inhibitors.

The SwissADME [16,17,18] and pkCSM [19] online web tools were used to predict the pharmacokinetic and toxicity properties of the designed compounds and FDA-approved direct oral anticoagulants, such as rivaroxaban, apixaban, edoxaban, and betrixaban (for chemical structures see Figure 8).

The designed thiourea and oxime ether ISV derivatives are composed of an isosteviol core (fused polycyclic region), and the part based on a phenyl or a five-membered heterocyclic ring. Therefore, the structural similarity of the analyzed compounds to other commercial drugs, i.e., rivaroxaban, apixaban, edoxaban, and betrixaban, is observed. This increases the likelihood of their activity as FXa inhibitors because the analyzed compounds contain additional (compared to ISV) structural fragments responsible for binding the molecule in the active site. These are, for example, five-membered heterocyclic rings present both in most of the designed compounds and the commercial drugs, e.g., thiophen and oxazole rings (as in rivaroxaban and apixaban) and phenyl rings (as in rivaroxaban, apixaban, and betrixaban). Molecular modeling shows that they are important for the interaction of the ligand with the active site [9,10]. The presence of halogen atoms attached to aromatic rings in most of the described relationships, as in rivaroxaban, edoxaban, and betrixaban molecules, is also important. It was proven that the halogen forms π interactions with the S4 pocket (Y99), which constitutes the key to the pharmacological activity of direct factor Xa inhibitors [3]. Chlorine atoms are especially responsible for these lipophilic interactions, which was also confirmed by QSAR analyses [9,10,20].

The SwissADME and pkCSM predictions showed that the newly designed ISV-like compounds possess promising predicted physiochemical, pharmacokinetic, and toxicity properties (Table 5). In comparison to marketed anticoagulants, the designed isosteviol derivatives are considered to be less soluble, more lipophilic, and characterized by a lower total clearance and a higher maximum recommended tolerated dose in humans. Moreover, isosteviol analogs are less likely to be cytochrome P450 inhibitors as well as be associated with disrupted normal functioning of the liver. Only one of all the evaluated molecules, i.e., E24, is not a substrate of P-glycoprotein (P-gp). For all evaluated molecules, the volume of distribution is small (typical for anticoagulants), they all are CYP3A4 substrates and are likely to be poorly distributed to the brain. So, they are devoid of central nervous system side effects.

It is well known that all representatives of NOACs are substrates of P-gp and thus, may interact with strong inducers or inhibitors of this drug transporter. Additionally, drugs that alter the cytochrome P450 isoenzyme (CYP) activities can also affect the efficacy of rivaroxaban and apixaban [21]. The ADMET results obtained in Table 5 confirm this phenomenon. It is worth mentioning that, although rivaroxaban has been associated with a low incidence of serum enzyme elevation during treatment, there have been rare cases of clinically evident liver injury with jaundice [22]. The other direct factor X inhibitors, such as apixaban and edoxaban, display a lower rate of hepatotoxicity. According to the abovementioned data, all the target compounds may be a potential alternative to NOACs. Taking into consideration the MD results mentioned above, the main attention is paid to the molecule denoted as E24, which complies with Lipinski’s rule of five (RO5) and possesses a predicted SwissADME bioavailability score of 0.55. Similar results were obtained for rivaroxaban, apixaban, and betrixaban. Interestingly, edoxaban does not obey the RO5. The important parameter for the lead-optimization process is synthetic accessibility. As shown in Table 5, it is theoretically easier to synthesize the FDA-approved FXa inhibitors than the designed isosteviol derivatives. Interestingly, edoxaban with the synthetic accessibility score of 5.04 is halfway from very easy (1) to very difficult (10). The synthetic accessibility score of studied ISV derivatives ranges between 6.63 and 7.14, which means that chemical synthesis may be challenging. However, taking into account promising predicted FXa inhibitory activity as well as ADMET parameters, it is worth taking action because novel isosteviol derivatives may become potential oral anticoagulants.

### 2.3. Limitations of the Present Study

This is a theoretical study conducted to prioritize some of the designed ISV-like compounds for future synthesis and biological evaluation. However, some impediments in translating it into actual practice may occur due to the limitations of the methods used. Using a single trajectory for each complex enabled the examination of multiple ISV complexes with FXa. However, additional repetitions of the trajectories could validate the results and provide additional insights. Additionally, MD studies are limited by the short time scales that are typically simulated because many biochemical processes, such as receptor conformational shifts, that are relevant to drug binding may occur on timescales that are much longer than those that can be simulated [23,24]. Moreover, in silico ADMET models also have limitations due to data availability and selecting suitable experimental data for prediction. These models rely on machine learning and molecular fingerprints to predict ADMET properties [25].

## 3. Materials and Methods

### 3.1. MD Simulation

All the MD simulations were done using the GROMACS 2023.2 package [26] with an AMBER ff99-SB-ILDN force field [27]. Structures obtained from previously published docking studies served as starting geometries for complexes of ISV derivatives with FXa [9,10]. Ligand parameterization was conducted using the Acpype 2022.6.6 [28], employing the GAFF2 force field and the BCC charge method. The complexes were protonated at pH 7.0 and neutralized by adding the appropriate number of ions. A cubic simulation box (7.21505 nm × 7.21505 nm × 7.21505 nm) with a solute–box distance of 1 nm and a TIP3P explicit water model were used [29]. The charge of the complex was neutralized by adding one Cl^-^ ion. The LINCS constraint algorithm was applied, enabling calculations with a 2 fs time step [30]. The energy minimization of the systems was carried out using the steepest descent minimization algorithm. The system was equilibrated for 1 ns in the NVT ensemble, followed by an additional 1 ns in the NPT ensemble. The temperature was maintained at 300 K using the V-rescale thermostat, and the pressure was held at 1 bar through coupling with the C-rescale barostat. For nonbonded interactions, a cutoff of 10 Å was used. The production was set at 100 ns.

The RMSD of the protein and ligand was calculated using gmx rms, with least-squares fitting of backbone atoms to the first structure from the MD simulation. Ligand clustering was done using the gromos algorithm in the GROMACS software and a cutoff of 1.0 Å, based on frames sampled every 100 ps. For hydrogen-bond calculations using VMD 1.9.3 software [31], a cutoff distance of 3.5 Å for the donor–acceptor pair and a threshold angle of 30° were applied. Hydrophobic interactions between the ligands and the FXa were assessed using gmx mindist, with a 4.5 Å cutoff for distances between heavy amino acid atoms and ligand heavy atoms.

### 3.2. ADMET Properties Prediction

The SwissADME [16,17,18] and pkCSM [19] online web tools were utilized for predicting the physiochemical, pharmacokinetic, and toxicity properties and the drug likeness of studied molecules.

## 4. Conclusions

This study demonstrates the ability of novel thiourea and ether oxime derivatives of isosteviol to inhibit factor Xa activity. These derivatives interact with FXa like FDA-approved drugs. MD simulations revealed that four out of the nine derivatives form stable complexes with FXa, exhibiting interactions with key amino acids crucial for the binding of drugs like edoxaban and betrixaban. Among the thiourea derivatives of ISV, compound E10, possessing a 4-(oxazol-5-yl)phenyl substituent, forms a stable complex with FXa, as indicated by the stable RMSD of the ligand throughout the simulation. For the oxime ether derivatives of ISV, stable complex formation was observed in compounds E15, E20, and E24, possessing 2,5-dichlorothiophen-3-yl, 5-(trifluoromethyl)furan-2-yl and 4-fluorothiophen-3-yl substituents, respectively. ADMET/drug-likeness screening was conducted to assess the pharmacokinetic parameters of the tested isosteviol derivatives. The integrated findings from molecular dynamics simulations and ADMET designate E24 as the most promising drug candidate. This molecule, along with the other three derivatives, can undergo chemical synthesis and biological testing in further studies.

## Figures and Tables

**Table 1:** Structures of the designed isosteviol-based FXa inhibitors, along with their IUPAC names and molar masses.

Ligand	Structure	IUPAC Name	Binding Free Energy [kcal/mol]	Molar Mass [g/mol]
E01	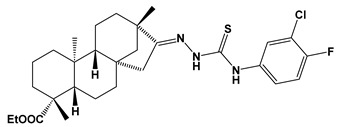	ethyl (4R,4aS,6aR,9S,11aR,11bS,E)-8-(2-((3-chloro-4-fluorophenyl)carbamothioyl)hydrazineylidene)-4,9,11b-trimethyltetradecahydro-6a,9-methanocyclohepta[a]naphthalene-4-carboxylate	−8.8	548.16
E04	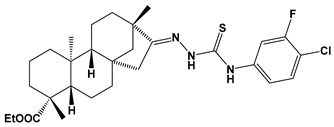	ethyl (4R,4aS,6aR,9S,11aR,11bS,E)-8-(2-((4-chloro-3-fluorophenyl)carbamothioyl)hydrazineylidene)-4,9,11b-trimethyltetradecahydro-6a,9-methanocyclohepta[a]naphthalene-4-carboxylate	−8.5	548.16
E10	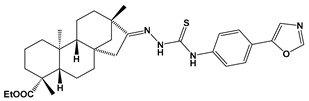	ethyl (4R,4aS,6aR,9S,11aR,11bS,E)-4,9,11b-trimethyl-8-(2-((4-(oxazol-5-yl)phenyl)carbamothioyl)hydrazineylidene)tetradecahydro-6a,9-methanocyclohepta[a]naphthalene-4-carboxylate	−8.8	562.77
E15	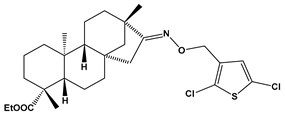	ethyl (4R,4aS,6aR,9S,11aR,11bS,E)-8-(((2,5-dichlorothiophen-3-yl)methoxy)imino)-4,9,11b-trimethyltetradecahydro-6a,9-methanocyclohepta[a]naphthalene-4-carboxylate	−8.3	526.56
E20	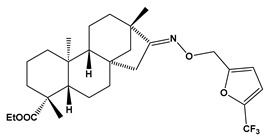	ethyl (4R,4aS,6aR,9S,11aR,11bS,E)-4,9,11b-trimethyl-8-(((5-(trifluoromethyl)furan-2-yl)methoxy)imino)tetradecahydro-6a,9-methanocyclohepta[a]naphthalene-4-carboxylate	−8.1	509.60
E21	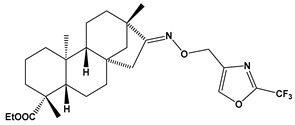	ethyl (4R,4aS,6aR,9S,11aR,11bS,E)-4,9,11b-trimethyl-8-(((2-(trifluoromethyl)oxazol-4-yl)methoxy)imino)tetradecahydro-6a,9-methanocyclohepta[a]naphthalene-4-carboxylate	−8.3	510.59
E23	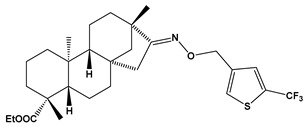	ethyl (4R,4aS,6aR,9S,11aR,11bS,E)-4,9,11b-trimethyl-8-(((5-(trifluoromethyl)thiophen-3-yl)methoxy)imino)tetradecahydro-6a,9-methanocyclohepta[a]naphthalene-4-carboxylate	−8.1	525.67
E24	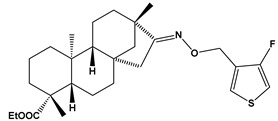	ethyl (4R,4aS,6aR,9S,11aR,11bS,E)-8-(((4-fluorothiophen-3-yl)methoxy)imino)-4,9,11b-trimethyltetradecahydro-6a,9-methanocyclohepta[a]naphthalene-4-carboxylate	−8.2	475.66
E25	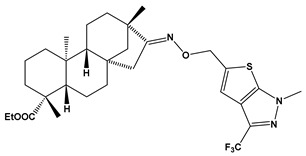	ethyl (4R,4aS,6aR,9S,11aR,11bS,E)-4,9,11b-trimethyl-8-(((1-methyl-3-(trifluoromethyl)-1H-thieno[2,3-c]pyrazol-5-yl)methoxy)imino)tetradecahydro-6a,9-methanocyclohepta[a]naphthalene-4-carboxylate	−8.2	579.72

**Table 2:** RMSD values of heavy ligand atoms for representative structures for each cluster. Docking ligand poses were utilized as reference structures for the analysis.

Docking Pose	Conformation from MD Simulation		
	First Cluster	Second Cluster	Third Cluster
E10	1.21 Å	-	-
E15	1.63 Å	0.44 Å	-
E20	1.06 Å	1.84 Å	1.67 Å
E24	0.45 Å	-	-

**Table 3:** Analysis of hydrogen bonds between FXa and ligands. Hydrogen-bond calculations employed a 3.5 Å cutoff for donor–acceptor distance and a 30° threshold for the angle. The hydrogen bonds that existed more than 10% of the time were analyzed.

Complexes	Hydrogen Bonds				
	Donor	Acceptor	Occupancy [%]	Distance ± SD [Å]	Angle ± SD [°]
E10	E10 (N3)	G216 (O)	22.42	3.00 ± 0.32	28.04 ± 11.27
E15	G216 (N)	E15 (N)	28.63	4.22 ± 0.85	20.37 ± 11.18
	Y99 (N)	E15 (O1)	26.23	9.60 ± 5.23	40.26 ± 23.62
E20	-	-	-	-	-
E24	G216 (N)	E24 (N)	40.10	3.56 ± 0.32	16.1 ± 7.93

**Table 4:** Hydrophobic interactions between the ligand and three key amino acid residues crucial for FXa-inhibitor binding. For quantifying the occupancy of hydrophobic interactions, a distance cutoff of 4.5 Å was applied between the heavy atoms of the ligand and the heavy atoms of the amino acid side chains.

Ligand	Hydrophobic Interactions		
	Y99	F174	W215
E10	96.94%	88.55%	99.89%
E15	99.56%	57.40%	99.74%
E20	99.96%	26.10%	99.98%
E24	99.01%	81.44%	99.98%

**Table 5:** Predicted physiochemical, pharmacokinetic, and toxicity properties for designed compounds and marketed direct oral anticoagulants (the most promising drug candidate, E24, is highlighted in yellow).

Compound	Molecular Weight	Num. Rotatable Bonds	Num. H-Bond Acceptors	Num. H-Bond Donors	TPSA [Å²] ^1^	Consensus Log Po/w	Lipinski	Bioavailability Score	Water Solubility [log mol/L]	Synthetic Accessibility	Intestinal Absorption [% Absorbed]	P-Glycoprotein Substrate	VDss ^2^ [log L/kg]	BBB Permeability ^3^ [log BB]	CYP2D6 Substrate	CYP2D6 Inhibitor	CYP3A4 Substrate	CYP3A4 Inhibitor	CYP1A2 Inhibitor	CYP2C19 Inhibitor	CYP2C9 Inhibitor	Total Clearance [log ml/min/kg]	AMES ^4^ Toxicity	Max. Tolerated Dose [log mg/kg/day]	Oral Rat Chronic Toxicity [0.644]	Hepatotoxicity
E01	548.16	7	4	2	94.81	6.69	No	0.17	−5.198	6.63	92.59	Yes	−0.211	−0.233	No	No	Yes	Yes	No	No	No	−0.711	No	−0.264	0.644	No
E04	548.16	7	4	2	94.81	6.70	No	0.17	−5.322	6.67	92.56	Yes	−0.202	−0.233	No	No	Yes	Yes	No	No	No	−0.712	No	−0.243	0.627	No
E10	562.77	8	5	2	120.84	6.01	No	0.17	−5.463	6.96	94.29	Yes	0.377	−0.086	No	No	Yes	No	No	No	No	−0.682	No	−0.02	0.356	No
E15	526.56	6	4	0	76.13	7.38	No	0.17	−5.792	6.78	94.58	Yes	−0.081	−0.13	No	No	Yes	No	No	No	No	−0.247	No	0.746	0.481	No
E20	509.60	7	8	0	61.03	6.52	No	0.17	−5.917	6.90	96.36	Yes	0.035	−0.195	No	No	Yes	No	No	No	No	−0.107	No	0.257	0.711	No
E21	510.59	7	9	0	73.92	5.96	No	0.17	−5.623	6.69	96.81	Yes	−0.19	−0.949	No	No	Yes	Yes	No	No	No	−0.093	No	0.129	0.806	No
E23	525.67	7	7	0	76.13	7.24	No	0.17	−5.673	6.91	94.42	Yes	−0.177	−0.12	No	No	Yes	No	No	No	No	−0.363	No	0.523	0.588	No
E24	475.66	6	5	0	76.13	6.39	Yes	0.55	−5.248	6.71	96.60	No	−0.232	−0.196	No	No	Yes	No	No	No	No	−0.249	No	0.643	0.972	No
E25	579.72	7	8	0	93.95	7.11	No	0.17	−5.509	7.14	94.72	Yes	−0.185	−0.281	No	No	Yes	Yes	No	No	No	−0.331	No	0.379	0.54	No
R ^5^	435.88	6	5	1	116.42	2.29	Yes	0.55	−4.382	3.63	92.80	Yes	−0.687	−1.022	No	No	Yes	Yes	No	Yes	Yes	0.296	Yes	−0.232	1.125	Yes
A ^6^	459.50	5	5	1	110.76	2.30	Yes	0.55	−4.181	3.48	88.96	Yes	−0.14	−0.985	No	No	Yes	Yes	No	Yes	Yes	0.247	No	−0.119	1.276	Yes
E ^7^	548.06	10	7	3	164.87	1.35	No	0.17	−3.377	5.04	72.09	Yes	−0.243	−1.082	No	No	No	Yes	No	No	No	0.474	No	0.109	2.471	Yes
B ^8^	451.91	9	5	3	107.41	3.22	Yes	0.55	−4.313	3.05	76.59	Yes	−0.08	−1.276	No	No	Yes	Yes	No	Yes	Yes	0.257	No	0.685	1.181	Yes

^1^ Topological polar surface srea; ^2^ the volume of distribution; ^3^ the blood–brain barrier permeability; ^4^ the Ames test; ^5^ rivaroxaban; ^6^ apixaban; ^7^ edoxaban; and ^8^ betrixaban.

## Data Availability

The data presented in this study are available within this article.
